# Selective Adsorption of Amino Acids in Crystals of Monohydrocalcite Induced by the Facultative Anaerobic *Enterobacter ludwigii* SYB1

**DOI:** 10.3389/fmicb.2021.696557

**Published:** 2021-07-29

**Authors:** Yanyang Zhao, Zuozhen Han, Huaxiao Yan, Hui Zhao, Maurice E. Tucker, Xiao Gao, Na Guo, Ruirui Meng, Daniel Cosmos Owusu

**Affiliations:** ^1^Shandong Provincial Key Laboratory of Depositional Mineralization and Sedimentary Minerals, College of Earth Science and Engineering, Shandong University of Science and Technology, Qingdao, China; ^2^Laboratory for Marine Mineral Resources, Qingdao National Laboratory for Marine Science and Technology, Qingdao, China; ^3^Department of Bioengineering, College of Chemical and Biological Engineering, Shandong University of Science and Technology, Qingdao, China; ^4^School of Earth Sciences, University of Bristol, Bristol, United Kingdom; ^5^Cabot Institute, University of Bristol, Bristol, United Kingdom

**Keywords:** biomineralization, amino acids, extracellular polymeric substances, monohydrocalcite, facultative anaerobic bacteria

## Abstract

The morphology, crystal structure, and elemental composition of biominerals are commonly different from chemically synthesized minerals, but the reasons for these are not fully understood. A facultative anaerobic bacterium, *Enterobacter ludwigii* SYB1, is used in experiments to document the hydrochemistry, mineral crystallization, and cell surface characteristics of biomineralization. It was found that carbonate anhydrase and ammonia production were major factors influencing the alkalinity and saturation of the closed biosystem. X-ray diffraction (XRD) spectra showed that calcite, monohydrocalcite (MHC), and dypingite formed in samples with bacterial cells. It was also found that the (222) plane of MHC was the preferred orientation compared to standard data. Scanning transmission electron microscopy (STEM) analysis of cell slices provides direct evidence of concentrated calcium and magnesium ions on the surface of extracellular polymeric substances (EPS). In addition, high-resolution transmission electron microscopy (HRTEM) showed that crystallized nanoparticles were formed within the EPS. Thus, the mechanism of the biomineralization induced by *E. ludwigii* SYB1 can be divided into three stages: (i) the production of carbonate anhydrase and ammonia increases the alkalinity and saturation state of the milieu, (ii) free calcium and magnesium ions are adsorbed and chelated onto EPS, and (iii) nanominerals crystallize and grow within the EPS. Seventeen kinds of amino acids were identified within both biotic MHC and the EPS of SYB1, while the percentages of glutamic and aspartic acid in MHC increased significantly (*p* < 0.05). Furthermore, the adsorption energy was calculated for various amino acids on seven diffracted crystal faces, with preferential adsorption demonstrated on (111) and (222) faces. At the same time, the lowest adsorption energy was always that of glutamic and aspartic acid for the same crystal plane. These results suggest that aspartic and glutamic acid always mix preferentially in the crystal lattice of MHC and that differential adsorption of amino acids on crystal planes can lead to their preferred orientation. Moreover, the mixing of amino acids in the mineral structure may also have a certain influence on the mineral lattice dislocations, thus enhancing the thermodynamic characteristics.

## Introduction

As one of the most extensive processes on the Earth’s surface, microbially induced carbonate precipitation (MICP) plays a key role in biogeochemical cycles of carbon, as well as influences global climate and seawater chemistry (Lowenstam and Weiner, 1989; Li et al., 2019). In recent years, more attention has been draw to this topic in view of its potential biological and engineering applications, including: (1) pretreatment of seawater Ca–Mg desalination (Arias et al., 2017; Ansari et al., 2020), (2) rust protection of underwater steel and self-repair of building materials (Achal et al., 2015; Bundur et al., 2017; Guo et al., 2019), (3) bioremediation of heavy metal polluted soils (Lian et al., 2006; Achal et al., 2012), (4) fixation of atmospheric CO_2_ (Sharma and Bhattacharya, 2010), and (5) bioprecipitation of free hazardous ions (Zhang et al., 2015; Lu et al., 2020; Shi C. et al., 2020; Shi J. et al., 2020; Zhao et al., 2020a; Wang et al., 2021; Zhang et al., 2021). Thus, an in-depth understanding of the bioprecipitation mechanism and the formation of the special structure of biominerals is of great importance for the biomimetic design and synthesis of functional materials. Not only that, MICP also helps to understand the close interactions between inorganic and organic worlds from a natural microperspective.

Many microorganisms can induce precipitation of carbonate minerals under suitable conditions (Lowenstam and Weiner, 1989). To elucidate the formation processes of microbial minerals, numerous culture experiments have been conducted using different species of bacteria, including cyanobacteria, halophiles, aerobic heterotrophs, sulfate-reducing bacteria, and archaea (Sánchez-Román et al., 2011; Krause et al., 2012; Benzerara et al., 2014; Qiu et al., 2017; He et al., 2021), while facultative anaerobic bacteria have rarely been studied. Depending on the various bio-activities and ambient solution conditions, the biominerals produced can be dramatically different. The Mg concentration and Mg/Ca molar ratio are two key chemical factors in extracellular bacterial carbonate precipitation (Sánchez-Román et al., 2011; Han et al., 2018). Calcite, vaterite, and aragonite are the dominant precipitates in Mg-free solutions (Zhuang et al., 2018; Li et al., 2019), while Mg-calcite (low Mg-calcite, high Mg-calcite, very high Mg-calcite), dolomite (ordered and disordered) (Deng et al., 2010; Qiu et al., 2017; Liu et al., 2019), and various hydro-Ca–Mg carbonates are mineral phases in Mg-rich solutions (Sanz-Montero et al., 2019; Zhao et al., 2020b). These biominerals usually exhibit complex morphologies, including spherulite, dumbbell, and cauliflower shape (Sánchez-Román et al., 2011; Qiu et al., 2017; Zhang et al., 2020). In addition to the surface morphology, the crystal constants of minerals could also be affected by bacterial activities (Zhuang et al., 2018; Zhao et al., 2019). The morphology and composition of intracellular products are also affected by particular microorganisms. There are nanospherical amorphous calcium carbonate (ACC) inclusions in various cyanobacteria (Benzerara et al., 2014; Li et al., 2016), chained bullet-shaped magnetosomes in magnetotactic bacteria (Li et al., 2010, 2020), and irregular Ca–Mg-bearing inclusions in *Bacillus* bacterial species (Zhuang et al., 2018; Zhao et al., 2019). Under the influence of a microorganism’s activities, the entire process of mineral formation, including nucleation, crystal growth, mineral phase transformation, orientation, and particle assembly, can all be significantly affected.

Therefore, the focus of MICP is how the microbes regulate the formation of bioproducts. Whether in the natural environment, in the laboratory, and/or in biomimetic studies, it is widely accepted that the key factor is extracellular polymeric substances (EPS), secreted by microbes (Cusack and Freer, 2008; Krause et al., 2012; Perri et al., 2018; Huang et al., 2019; Popall et al., 2020; Yin et al., 2020; Zhao et al., 2020c). It has been demonstrated that negatively charged EPS could serve as nucleation sites; this is mainly caused by the deprotonation of carboxyl components (e.g., amino acids) under high pH conditions (Katz et al., 1998; Deng et al., 2010). Among the complex polymorphs of EPS, amino acids have always been regarded as the important source of organic carboxyl groups (Qiu et al., 2017). Qiu et al. (2017) focused on the amino acids in the surface layer (S-layer) of EPS and proposed that acidic amino acids contribute to the dehydration of Mg[H_2_O]_6_^2+^, leading to the formation of dolomite. After that, a series of microbial and bionic experiments further revealed that amino acids promote the nucleation of carbonate minerals on the EPS surface (Zhuang et al., 2018; Liu et al., 2019; Zhao et al., 2019). Molecular-leveled insight into amino acid interactions with mineral surfaces is vital to understand the nucleation and growth mechanisms of biomineralization. However, the role of amino acids from EPS in the regrowth and crystal orientation is seldomly explored. In general, the morphology and phase of biominerals are complex and diverse. Heretofore, in spite of several formation hypotheses (Qiu et al., 2017), the roles of amino acids in biomineral nucleation and growth are not fully understood, and the formation mechanisms of complex biomineral morphologies and crystal structure are still unclear.

To fill these gaps and provide details on these processes, a facultative anaerobic bacterial strain was separated from the river sediment and used in experiments of various Mg/Ca molar (Ca = 0.01 M) ratios. In this paper, the microbiological influence has been characterized in terms of identifying the change in hydrochemical parameters with changes in the medium. On the basis of a detailed discussion of mineral crystal physics (the crystallinity, micromorphology, organic functional group, amino acid composition, and thermal stability), this study focuses on organic compounds in EPS and biominerals, especially the role of amino acids in mineral nucleation and growth. Through density functional theory (DFT), the adsorption energy and characteristics of amino acids on the crystal faces were calculated. This may provide new insights into the microbial-induced carbonate polymorphism problem and the effect of organic compounds, especially amino acids, on carbonate nucleation and growth.

## Materials and Methods

### Materials

All of the inorganic reagents are of analytical grade. The beef extract, yeast extract, and tryptone are of biotech grade. All were purchased from Sinopharm Chemical Reagent Co., Ltd., Shanghai, China and also used as received. Deionized water was used in all experiments.

### Isolation, Purification, Identification, and Culture of *E. ludwigii* SYB1

Twenty grams of sediments from the Moshui River (36.002086°N, 120.120066°E), Shandong Province, China, were transferred to a conical flask with 150 ml autoclaved enrichment medium [0.52 g L^–1^ K_2_HPO_4_, 1.02 g L^–1^ NH_4_Cl, 0.13 g L^–1^ CaCl_2_, 2.02 g L^–1^ MgSO_4_⋅7H_2_O, 1.04 g L^–1^ yeast extract, 6 ml L^–1^ of 60% (weight percent) sodium lactate, 0.53 g L^–1^ Na_2_SO_4_, 0.51 g L^–1^ Fe(NH_4_)_2_(SO_4_)_2_, 0.52 g L^–1^ ascorbic acid, and 0.51 g L^–1^ cysteine]. After that, 20 ml of liquid paraffin was covered on the medium surface to create a relatively anaerobic environment and then allowed to stand in the anaerobic incubator at 30°C. After 48 h, the turbid enrichment medium was diluted 10^–5^-fold by cooled boiled deionized water, and then, 20 μl diluent was spread evenly on the solid medium. The components of the solid medium were the same as those in the enrichment medium, with an additional 20 g/L of agar. Once single colonies could be observed, the best growing strain was selected and purified 10 times; the dish sandwich anaerobic culture was used for the purification process. Eventually, a bacterium named SYB1 was purified and conserved at −20°C in 30% glycerol water solution.

The genomic DNA of SYB1 was extracted with the improved cetyltrimethylammonium bromide (CTAB) method and used as the template for polymerase chain reaction (PCR) with universal primers F27 (5′-AGAGTTTGATCCTGGCTCAG-3′), R1492 (5′-GGTTACCTTGTTACGACTT-3′). The PCR products were checked by agarose gel electrophoresis; a light band (approximately 1,500 bp in length) appeared and was recognized as the 16S rDNA fragment. After that, it was sequenced by the sequencing center of Shanghai Sangon Biotech Co., Ltd. (Shanghai, China). The sequences were assembled with DNAMAN 8.0. The complete 16S rDNA sequence was uploaded to GenBank of the National Center for Biotechnology Information (NCBI), and the Basic Local Alignment Search Tool (BLAST) program was applied to obtain the results of the 16S rDNA homology. A multiple sequence alignment was erected with the ClustalX software (version 2.0), and a phylogenetic tree of SYB1 was constructed with MEGA 6.0 using the neighbor-joining method. A series of physiological and biochemical experiments were also conducted to further confirm the taxonomy of SYB1.

As a facultative anaerobic bacterium, it was found that SYB1 grew better in the aerobic Nutrient Broth (NB) medium. Thus, NB medium (5 g L^–1^ beef extract, 10 g L^–1^ tryptone, and 5 g L^–1^ NaCl, pH 7.2) was used for liquid seed preparation. Once the optical density (OD) value measured at a wavelength of 600 nm reached 1.0, the preparation of the liquid seed was terminated.

### Biomineralization Experiments

The medium used for the biomineralization experiment was NB medium with additional Ca^2+^ and Mg^2+^ (Table 1). Dissolved CaCl_2_ and MgSO_4_⋅7H_2_O were added into NB medium by filtration sterilization through a 0.22-μm microporous membrane. The final Ca^2+^ concentration was fixed to be 0.01 M, while the Mg/Ca molar ratio was adjusted to be 0, 3, 6, and 9, respectively. The initial pH of the medium was adjusted to be 7.2 using NaOH solution. One hundred twenty milliliters of the experiment medium was aerobically inoculated with 1.2 ml (1%) of the prepared liquid seed (OD_600_ = 1) in a vertical flow clean bench. The controls include abiotic experiments (media without inoculation of bacteria) and biomimetic experiments (ionic species detected in the biotic vials were manually added to the media at the same rate). All of the groups were made in triplicates and were cultured at 37°C with constant shaking at 130 rpm.

**TABLE 1:** The components in the medium prepared for mineralization experiments.

Batch	MgSO_4_ (mol⋅L^–1^)	CaCl_2_ (mol⋅L^–1^)	NaCl (wt ‰)	Liquid seed (vt %)	Distilled H_2_O (ml)	Initial pH	Final mineralogy
1	0	0.01	5	1.5	100	7.2	Calcite
2	0.03	0.01	5	1.5	100	7.2	MHC
3	0.06	0.01	5	1.5	100	7.2	MHC, DYP
4	0.09	0.01	5	1.5	100	7.2	MHC, DYP

### Chemical Determination of Culture Medium

During the incubation period, the culture medium is sampled regularly to record chemical changes. The concentrations of Ca^2+^ and Mg^2+^ were measured by an atomic absorption spectrophotometer (AAS, TAS-986, China). The concentration of ammonium (NH_4_^+^) in the medium was measured by salicylic acid ammonium spectrophotometry national standard method. The color reagent is a mixture of salicylic acid solution and Seignette salt. NH_4_^+^ can react with salicylic acid and ClO^–^ to form blue compounds. Then, the concentration of NH_4_^+^ could be calculated according to the linear relationship between absorbance and concentration (Zhuang et al., 2018). The carbonate anhydrase (CA) activity was measured based on the catalysis of *p*-nitrophenylacetate to nitrophenol (Pocker and Stone, 1967; Smith and Ferry, 1999). The concentrations of carbonate and bicarbonate were determined by the national standard titration method. Color changing reactions were disclosed by the reaction of phenolphthalein and methyl orange with bacterial fermentation liquid (Zhuang et al., 2018; Zhao et al., 2020b). To determine the influence of bicarbonate and carbonate concentrations in the pH increase process, the simulated solution with the same concentrations of bicarbonate and carbonate in the medium was prepared, and pH was measured using a pH meter.

### Mineral Harvest and Sample Characterization

The cultures were checked periodically until the appearance of biominerals, and the minerals were then harvested by settlement after ultrasonic vibration, washed three times with absolute alcohol, and dried at room temperature for 48 h. X-ray diffraction (XRD) patterns of the products were recorded on a Japan D/Max-RC X-ray diffractometer with a 2θ angle range of 10–60°, a step size of 0.02, and a count time of 8°min^–1^. Morphologies of the biominerals were investigated by a Japan Hitachi S-4800 field emission scanning electron microscope (FESEM) at a voltage of 5.0 kV, and element information was collected using a Japan EX-450 energy dispersive spectrometer (EDS). The Fourier transform infrared spectroscopy (FTIR) spectra were collected using an American Nicolet 380 in an atmospheric background with a scanning wavenumber range of 5,000–400 cm^–1^ by the KBr pellets method. High-resolution transmission electron microscopy (HRTEM) and selected area electron diffraction (SAED) were conducted on a Japan JEM-2100 microscope at an accelerating voltage of 200 kV. X-ray photoelectron spectra (XPS) were recorded on a Thermo ESCALAB 250XI photoelectron spectrometer with Al *K*α radiation, over the binding energy range of 0–1,350 eV. The biominerals (0.2 g) were observed by a laser scanning confocal microscope (LSCM, Olympus FV1000-SIM, Olympus, Tokyo, Japan) at an excitation wavelength of 650 nm after being labeled by 5 ml Cy5 NHS ester (a reactive dye for the labeling of amino groups in peptides, proteins, and oligonucleotides) dimethyl sulfoxide (DMSO) solution (0.05 M) for 4 h. Ultrathin slices of SYB1 cells were made according to the published method (Zhao et al., 2020b) and were also investigated by HRTEM, STEM, and SAED with the same experimental conditions noted above.

The precipitates in the biomimetic experiments were also analyzed by SEM at the same conditions. To compare the difference between biotic and chemically synthesized monohydrocalcite (MHC), the MHC was also synthesized in inorganic solution (initial concentrations: 0.03 mol⋅L^–1^ MgCl_2_, 0.01 mol⋅L^–1^ CaCl_2_, 0.015 mol mol⋅L^–1^ Na_2_CO_3_) according to the published method (Kitajima et al., 2020). Thus, the formation hydrochemical conditions of chemically synthesized MHC were similar to those of the biotic MHC. After that, thermogravimetric and differential scanning calorimetry (TG-DSC) was conducted employing a TGA/DSC1/1600LF thermal analyzer (METTLER TOLEDO Co., Switzerland) with different heating rates (5, 10, 20, and 30°C⋅min^–1^) from room temperature to 1,000°C under the protection of a pure nitrogen flow.

### EPS Extraction and Amino Acids Determinization

Extracellular polymeric substances were extracted by a modified heating method reported by Morgan et al. (1990). Cells of *E. ludwigii* SYB1 in the stationary phase were collected by centrifugation at 2,500 rpm for 15 min. After that, the precipitated cells were washed with the Hank’s balanced salt solution (per liter: NaCl, 8.01 g; KCl, 0.4 g; CaCl_2_, 0.14 g; NaHCO_3_, 0.35 g; KH_2_PO_4_, 0.06 g; glucose, 0.34 g; pH 7.2) for three times. Then, the cells were suspended in Hank’s balanced salt solution (HBSS) solution and cultured in a water bath at 60°C for 40 min, then centrifuged at a speed of 10,000 rpm for 5 min. The supernatant was vacuum dried in a freeze dryer at −60°C for 24 h. Finally, the dried EPS powder was analyzed by an amino acid analyzer (L-8900, Hitachi, Tokyo, Japan). The biominerals were washed three times after they were soaked in deionized water for 1 day. The supernatant was scanned using FTIR to confirm that the absorbed organic matters on surfaces were totally removed. After the minerals were dissolved by hydrochloric acid (0.05%), the solution was also vacuum dried in a freeze dryer at −60°C for 24 h. The dried powder was also analyzed by the same amino acid analyzer.

### Data Processing

#### Calculation of Saturation Index for the MHC

Based on the hydrochemical data, the saturation index for the MHC was calculated by the PHREEQC (version 3) program. The dissolution reaction of MHC used in the program is ${\text{CaCO}}_{3}\cdot {\text{H}}_{2}\text{O}+{\text{H}}^{+}↔{\text{HCO}}_{3}^{-}+{\text{Ca}}^{2+}+{\text{H}}_{2}\text{O}$, log K at 25°C for the reaction is 2.73, enthalpy for the reaction at 25°C is −20,470 J⋅mol^–1^.

#### Calculation of Adsorption Energy

In this work, the DFT calculation was performed using the Dmol^3^ program (Perdew et al., 1996). The exchange-correlation interaction was treated by the generalized gradient approximation (GGA) with PBE functional (Delley, 2000). A double numerical quality basis set with a d-type polarization function (DNP) (Delley, 1990) was utilized for all the geometric optimizations and total energy calculations. The core electrons were modeled using effective core pseudopotentials (ECPs) by Dolg et al. (1987) and Bergner et al. (1993). All calculations were spin unrestricted. The positions of all the atoms were fully relaxed until the following convergence criteria were met, respectively: 0.002 Ha/Å for force, 10^–5^ Ha for total energy, and 0.005 Å for displacement. The real space cutoff radius was 4.1 Å. The self-consistent field computations criterion was chosen to be 10^–6^ Ha. The adsorption energy (*E*_*ad*_) is defined as: *E*_*ad*_ = *E*_*Surf* + *Adsorbate*_ − *E*_*Adsorbate*_ − *E*_*Surf*_, where *E*_*Surf* + *Adsorbate*_ is the total energy of a selected crystal face adsorbed with amino acids, *E*_*Adsorbate*_ is the total energy of amino acids, and *E*_*surf*_ is the total energy of the crystal face.

#### Refinement of XRD Data and Thermotic Kinetic Calculation

Based on the XRD data, the mineral compositions were calculated by the Rietveld refinement method (Han et al., 2014). The thermal decomposition mechanism and kinetic parameters of monohydrocalcites were calculated and verified by Kissinger–Akahira–Sunose (KAS) and Flynn–Wall–Ozawa (FWO) methods (Kissinger, 1957; Flynn and Wall, 1966).

## Results

### Identification of the Strain

**FIGURE 1:**
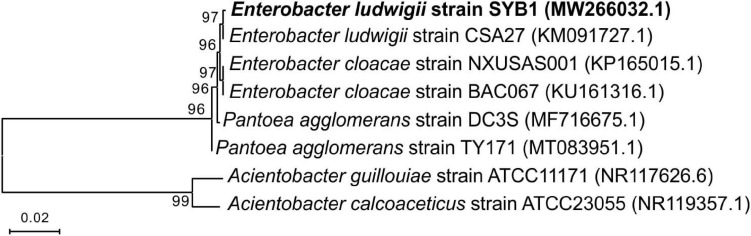
Phylogenetic tree of *Enterobacter ludwigii* SYB1 and its closest relatives based on 16S rRNA sequence, which was constructed by the neighbor-joining (NJ) method.

The fully developed single colony of the strain SYB1 grown on solid NB medium at 37°C for 24 h was moist, round in shape, and light yellow in color. Furthermore, SYB1 also showed positive results for citrate, indole, V-P, and the nitrate reduction test (Supplementary Table 1 and Supplementary Figure 1). A 1,443-base-pair (bp) fragment of the 16S rRNA gene was amplified from the strain SYB1. The complete 16S rDNA sequence was uploaded to GenBank of the NCBI and received an accession number of MW266032.1. The BLAST matching analysis showed that the 16S rDNA gene sequence of the strain had a high similarity (99%) to that of *E. ludwigii*. The phylogenetic tree was constructed by the neighbor-joining (NJ) method using MEGA 6.0 software, and it clearly showed that the strain SYB1 clustered with members of *E. ludwigii* strains (Figure 1). With these features, the strain was identified as *E. ludwigii* SYB1.

### Growth of *E. ludwigii* SYB1 and Hydrochemistry in the Medium

**FIGURE 2:**
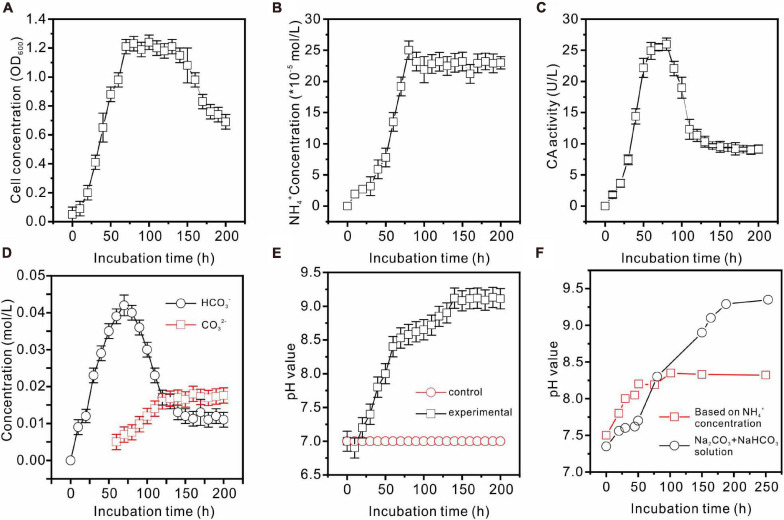
Biochemical characteristics of *E. ludwigii* SYB1 strain in medium without Ca^2+^ and Mg^2+^. **(A)** Growth curve of *E. ludwigii* SYB1; **(B)** concentration of NH_4_^+^; **(C)** CA activities; **(D)** concentrations of HCO_3_^–^ and CO_3_^2–^; **(E)** pH values of experimental and control groups; **(F)** pH values calculated based on NH_4_^+^ concentration and detected pH values of NaHCO_3_ + Na_2_CO_3_ solution.

*Enterobacter ludwigii* SYB1 grew well in all of the experimental NB media with Mg/Ca molar ratios of 0, 3, 6, and 9. The growth curve of *E. ludwigii* SYB1 in NB media exhibits four stages: the lag growth stage, the logarithmic stage, the stable stage, and the decline stage (Figure 2A). In the lag growth stage (0–10 h), the cell concentration showed almost no change, while the pH decreased from 7.2 to 6.76 during this stage due to the acidification of the media, provoked by the hydration of CO_2_ (Figures 2A,B). The range of the logarithmic phase was from 10 to 70.4 h, and there was an increase in OD_600_ value (from 0.1 to 1.2), while the NH_4_^+^ concentration (2.426 × 10^–4^ mol⋅L^–1^), HCO_3_^–^ concentration (0.042 mol⋅L^–1^), and CA activity (25.2 U⋅L^–1^) reached their maximum values (Figures 2B–D) at about 70–80 h. The third phase was the stationary phase in a time range of 60–150 h; the cell concentration was stable during this time (OD_600_ = 1.2), and the NH_4_^+^ concentration was basically unchanged (Figure 2B). After 150 h, the growth of SYB1 entered into the decline phase when the cell concentration decreased gradually because of the shortage of nutrients and the accumulation of metabolic products. The calculated pH value based on the concentration of ammonium suggested that ammonium production was able to adjust the pH to around 8.2 (Figure 2F). However, the detection of pH based on the concentration of sodium carbonate and sodium bicarbonate indicated that CA promoted the pH increasing to about 9.2 (Figures 2E,F). The pH curve of the mixed Na_2_CO_3_ and NaHCO_3_ solution based on the HCO_3_^–^ and CO_3_^2–^ concentrations catalyzed by CA (Figure 2F) showed that pH values were higher than those calculated according to the NH_4_^+^ ion concentration, suggesting that the HCO_3_^–^ and CO_3_^2–^ ions produced by the combination effect of CA and ammonia may have played an important role in pH increase.

### Mineralogy of Carbonates Induced by *E. ludwigii* SYB1

In the control groups, no precipitates were presented in the abiotic experiments. In the biomimetic groups, white precipitates appeared immediately once the sodium carbonate solution was added into the flask (corresponded to about 50 h after incubation of the biotic experiment). In the Mg-free medium, the total amount of white precipitates is the largest. It was also found that mainly rhombohedral and spherulitic calcite was formed (Supplementary Figure 2A). With the increasing magnesium concentration in the medium, the volume of precipitates decreases significantly. It was also found that in the medium with a high Mg/Ca molar ratio (Mg/Ca = 3, 6, and 9), the precipitates were always remained in the amorphous state and cannot be transformed into crystalline minerals after 14 days (Supplementary Figures 2B–D).

**FIGURE 3:**
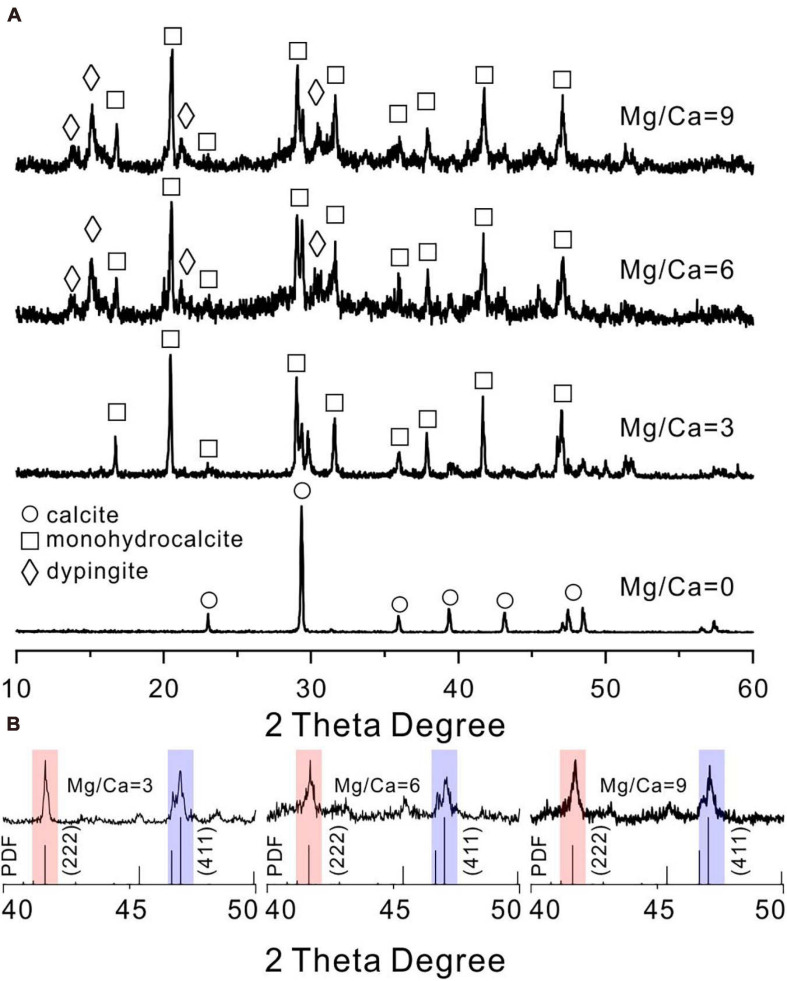
X-ray diffraction (XRD) patterns of carbonates mediated by *E. ludwigii* SYB1 in Nutrient Broth (NB) media. **(A)** XRD patterns of biominerals in medium with various Mg/Ca molar ratios (Ca = 0.01 M). **(B)** The intensity of (222) crystal face (red background) of microbial monohydrocalcite (Mg/Ca = 0, 3, and 6) showing a significant increase compared to (411) face (blue background).

In contrast, when cultured for 14 days in biotic experiments, it could be determined that calcite, monohydrocalcite (MHC, CaCO_3_⋅H_2_O), and dypingite [DYP, Mg_5_(CO_3_)_4_(OH)_2_⋅5H_2_O] were induced by *E. ludwigii* SYB1 according to the XRD data (Figure 3A). Both the mineral phases and their relative abundances changed with Mg/Ca molar ratios in the medium. Calcite was the only mineral phase in the bioprecipitates harvested from the medium without magnesium. With an Mg/Ca ratio of 3, MHC became the major mineral phase. MHC (73.25%) and DYP (26.75%) were dominant phases in the culture medium with an Mg/Ca ratio of 6. The mineralogy in the medium with an Mg/Ca ratio of 9 was the same as that in the medium with an Mg/Ca ratio of 6, although the percentage of MHC decreased to 69.79% and DYP increased to be 30.21%. The decrease in MHC percentage may be related to the decrease in its saturation index. The maximum saturation index of MHC was about 1.94, which occurred at about 100 h after incubation in the medium with the Mg/Ca molar ratio of 3. As the ratio of Mg/Ca increases, the saturation index of MHC has a downward trend, and they were 1.74 and 1.61 in the medium with the Mg/Ca molar ratio of 6 and 9, respectively. This was consistent with the decrease in MHC weight percentage reflected in the XRD data. After crystal refinement, it was found that the relative intensity of MHC (formed in a medium with Mg/Ca ratio of 3, 6, and 9) crystal face (222) was much higher than the standard data, indicating a preferred orientation along (222) (Figure 3B). The patterns at an Mg/Ca ratio of 6 and 9 seemed to be rougher compared to that in other groups, which was caused by the existence of weak crystallized flakey DYP. These results demonstrated that *E. ludwigii* SYB1 was able to mediate the formation of various carbonate minerals. The variation in Mg/Ca molar ratio from 0 to 9 does affect the biomineral phase, and the crystal structure was also distorted with the influence of the bacteria.

**FIGURE 4:**
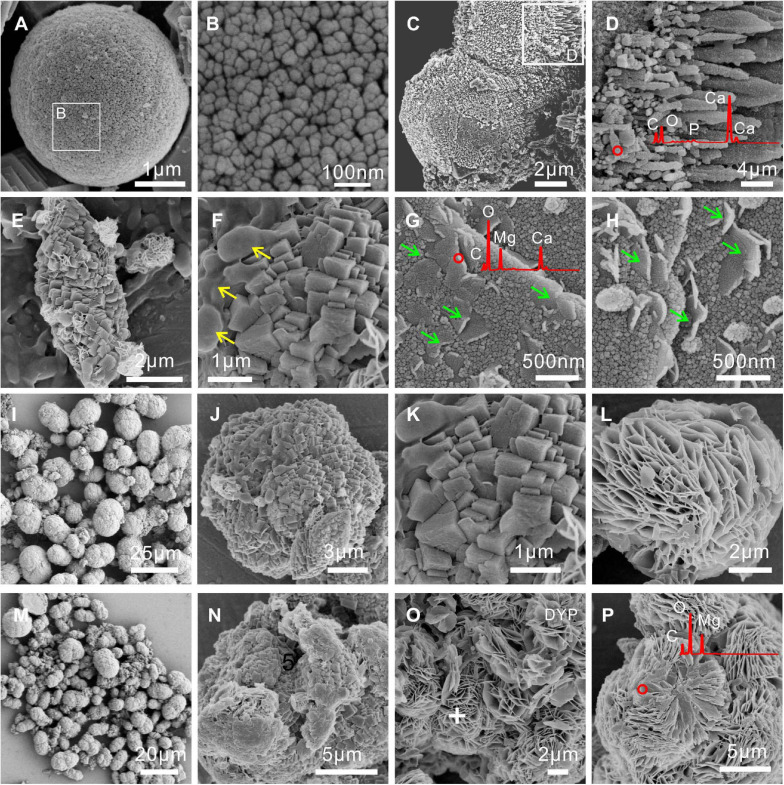
Morphologies and elemental composition of biominerals induced by *E. ludwigii* SYB1 in Nutrient Broth (NB) media with different Mg/Ca molar ratios. Samples in medium with **(A–D)** Mg/Ca ratio of 0 and **(E–H)** Mg/Ca ratio of 3. The cells and extracellular polymeric substances (EPS) on the mineral surface were marked by the yellow arrow; a layer of high Mg concentration coating on the surface of monohydrocalcite (MHC) was marked by the green arrow. Samples in medium with **(I–L)** Mg/Ca ratio of 6 and **(M–P)** Mg/Ca ratio of 9, respectively.

FESEM images and EDS analyses of biominerals mediated by *E. ludwigii* SYB1 are shown in Figure 4. Compared to carbonate minerals synthesized via the purely chemical method, the morphologies of the biominerals obtained were more complex. Chemical calcite was usually hexagonal rhombohedral (Zhuang et al., 2018), while there were spherulitic and dumbbell-shaped calcite particles with rough and finger-like surfaces in precipitates induced by *E. ludwigii* SYB1 in the Mg^2+^ free medium (Figures 4A–D). A coating of high Mg concentration was detected on the surface of MHC in the medium with an Mg/Ca molar ratio of 3 (marked by green arrow) (Figures 4E–H). It has been reported that chemical low-Mg MHC usually consists of nanoparticulate crystallites that are aggregated to form elongated particles (Rodriguez-Blanco et al., 2014), while it always exhibited the two connected spherules with a diameter about 8–9 μm in the medium with a high Mg/Ca ratio of 6 and 9 (Figures 4I–P). There was also a flower-like flaky DYP in the bioprecipitates (Figures 4L,O,P); a similar mineral assemblage has been reported from a natural alkaline lake at central Spain by Sanz-Montero et al. (2019). Numerous bacterial cells and EPS could also be recognized on the mineral surface (marked by a yellow arrow).

The FTIR spectrum shows the characteristic vibrational bands of various biominerals (Supplementary Figure 3). Since most minerals (calcite, MHC, and DYP) contained an abundance of carbonate groups, each spectrum showed vibrations of the carbonate ion including ν_4_ in-plane bending located at ∼725 cm^–1^, ν_2_ out-of-plane bending located at ∼874 cm^–1^, and ν_3_ antisymmetric stretching located at ∼1,409 cm^–1^. In our results, the ν_4_-band showed a weak intensity, while the ν_3_- and ν_2_-bands are strong and sharp. There were also lattice vibrations of MHC (∼1,068 cm^–1^) (Wang Y. et al., 2015) and DYP (948 and 1,008 cm^–1^) (Frost et al., 2008). In addition, some bands belonging to organic compounds could also be identified, including carboxyl C = O (1,800, 1,720, 1,650, and 1,406 cm^–1^), C–N (peptide bonds vibration approximately 1,260 cm^–1^), COO^–^ (approximately 1,659 cm^–1^), C–O–C (asymmetric stretching, 1,225 cm^–1^), and N–H (1,648 cm^–1^) can be also detected out (Zhuang et al., 2018). Some weak absorption peaks in the range of 1,200–850 cm^–1^ were ascribed to the vibration of C–O–C (glycosidic linkage), C–C, and C–O–H from carbohydrates and amino acids. It should also be noted that many organic functional groups were overlapped by strong bands from the minerals. Based on these results, it can be further confirmed that there are organic components within these biominerals.

### Detailed Characterization of MHC Induced by *E. ludwigii* SYB1

**FIGURE 5:**
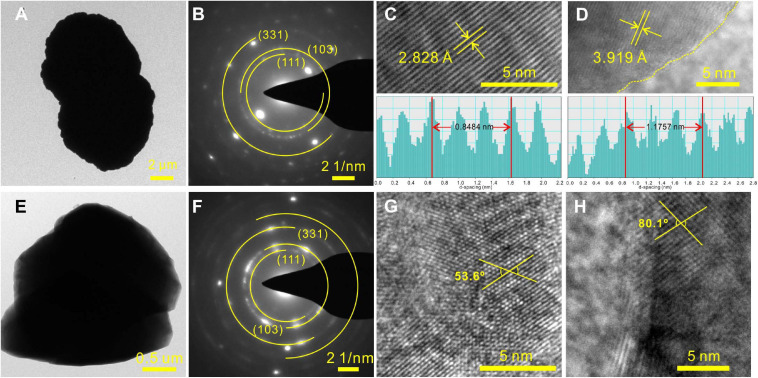
Representative high-resolution transmission electron microscopy (HRTEM) results of monohydrocalcite (MHC) induced by *E. ludwigii* SYB1 in Nutrient Broth (NB) media with Mg/Ca molar ratio of 3. **(A–D)** Morphology and selected area electron diffraction (SAED) patterns of MHC with two connected spherules structure. **(E–H)** Morphology and SAED patterns of irregular MHC.

Since MHC was always presented in all Mg^2+^-bearing NB media, it was selected as the typical biomineral and was analyzed in-depth. TEM, SAED, TG-DSC, LSCM, and XPS were used to characterize the MHC crystals obtained in the NB medium with an Mg/Ca ratio of 3. Figure 5 depicts the TEM images and representative SAED patterns of MHC with dumbbell and irregular morphologies. As shown in Figures 5A–D, the corresponding SAED pattern of a dumbbell crystal showed diffraction rings with well-developed spots. The lattice fringes also displayed interplanar spacings of 2.828 and 3.919 Å in the particle, which matched well, respectively, with those of the (301) and the (201) planes of the MHC crystal. In the irregular MHC (Figures 5E–H), the measured intersection angle between (222) and (401) was 53.6°, which was different from the actual angle, indicating that there were dislocations present. A similar situation occurred in the intersection angle between (221) and (003). This maybe the result of the mixture of impurity ions (Mg^2+^) and organic compounds. The increase in thermodynamic stability caused by structural adjustment was confirmed by our TG-DSC experiments (Supplementary Figure 4) and published literature (Zhuang et al., 2018; Liu et al., 2019).

The high-resolution XPS spectra of MHC induced by *E. ludwigii* SYB1 in the NB medium with an Mg/Ca molar ratio of 3 showed the peaks of C, O, Ca, Mg, N, and P on the surface composition and structure of the analyzed sample (Supplementary Figure 5). The Ca 2p core-level spectrum has two peaks, namely, Ca 2p 1/2 (350.5 eV) and Ca 2p 3/2 (347.2 eV) (Yuan et al., 2011). The Mg 1s core-level spectrum has a weak peak located at 1,304.5 eV, corresponding to the Mg native oxide state (Yuan et al., 2011). To further clarify the organic molecules involved in biomineralization, high-resolution scans of C 1s, O 1s, N 1s, P 2p, and S 2p were deconvoluted, and the corresponding functional groups were recognized. The C 1s peak was resolved into three peaks, including the peak at 284.5 eV corresponding to the C–(C,H) from amino acid side chains and the peak at 286.2 eV to C–N or (C = O)–N–C. The peaks located at 289.1 eV come from carbonate minerals (Yuan et al., 2011). The O 1s peak located at 531.2 eV can be attributed to carbonate in minerals (O = C–O); another O 1s peak at 533.06 eV is associated with (C = O)–OH (Yuan et al., 2011). The N 1s peak at 399.2 eV corresponds to C–NH_2_ and (C = O)–NH–C (Yuan et al., 2011).

**FIGURE 6:**
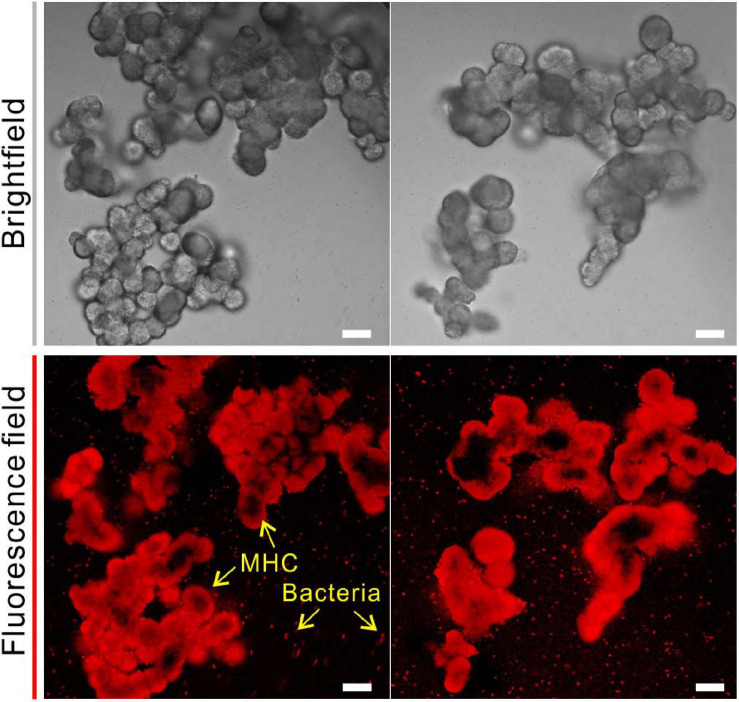
Confocal laser scanning microscope (CLSM) analysis of monohydrocalcite (MHC) crystals in medium with Mg/Ca ratio of 3 after labeling with Cy5 NHS ester. **(Top)** Brightfield of MHC. **(Bottom)** Fluorescence field.

In addition to the bulk analyses, the fluorescent labeling by CY5-NHS ester provided a direct evidence of amino groups bearing organic compounds (amino acids, peptides, proteins, oligonucleotides, etc.) in biotic MHC crystals. As shown in Figure 6, biotic MHC shows the spheroidal aggregates in bright field, which is consistent with the results of SEM. In the fluorescent field, it was easy to see that all MHC crystal particles have a red fluorescence signal, and some remaining bacterial cells have also been marked. However, it was noted that the fluorescence on the mineral surface seems to be emitted unevenly, indicating that the distribution of amino groups bearing organic materials in MHC crystals was also unevenly spaced.

**FIGURE 7:**
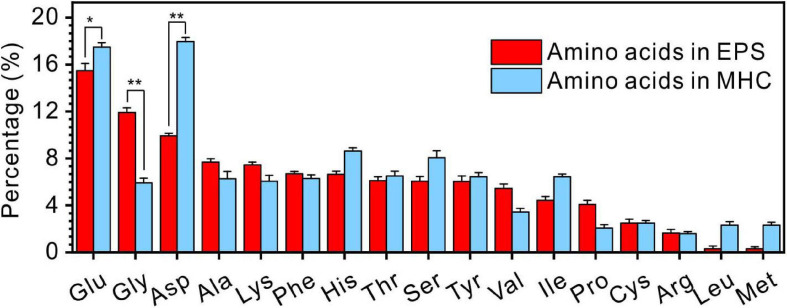
The comparison of amino acids in extracellular polymeric substances (EPS) extracted from *E. ludwigii* SYB1 and monohydrocalcite (MHC) in Nutrient Broth (NB) medium with an Mg/Ca molar ratio of 3. *p < 0.05, **p < 0.01.

It was also found that the kinds of amino acids in MHC induced by *E. ludwigii* SYB1 were the same as those in EPS of SYB1 (17 types of amino acids including Glu, Gly, Asp, Ala, Lys, Phe, His, Thr, Ser, Tyr, Val, Ile, Pro, Cys, Arg, Leu, and Met) (Figure 7). With regards to the content of various amino acids, Glu (15.3%) and Gly (17.6%) were the two most abundant amino acids in EPS. In contrast, Glu (17.26%) and Asp (17.35%) were the two most abundant amino acids in biotic MHC. On the one hand, this proves that the amino acids in MHC crystals were derived from bacterial EPS because they were charged in high pH (9.2) states. On the other hand, it suggests that different types of amino acids have different efficiencies in their inclusion into the crystal structure.

Taken together, combined with the above results, it is thus demonstrated that various amino acids from EPS are mixed into the crystallization and growth process of biominerals, and they do play an important role in the regulation of the crystal structure, morphology, and chemical properties of the minerals.

### Cells of *E. ludwigii* SYB1 in the Biomineralization Experiments

**FIGURE 8:**
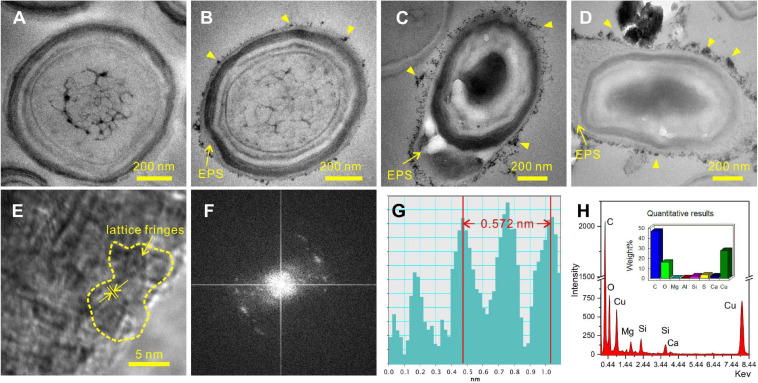
Transmission electron microscopy (TEM) images of 40-nm thin sections of *E. ludwigii* SYB1 cells. **(A)** Cells in seed liquid as control. **(B–D)** Cells in biomineralization experiments with Mg/Ca molar ratio of 3, 9, and 12. **(E)** Selected area electron diffraction (SAED) patterns of particles on the extracellular polymeric substances (EPS) in **(D)**. **(F,G)** Fast Fourier transform (FFT) pattern of selected area in **(E)**. **(H)** Elemental composition of the particles.

**FIGURE 9:**
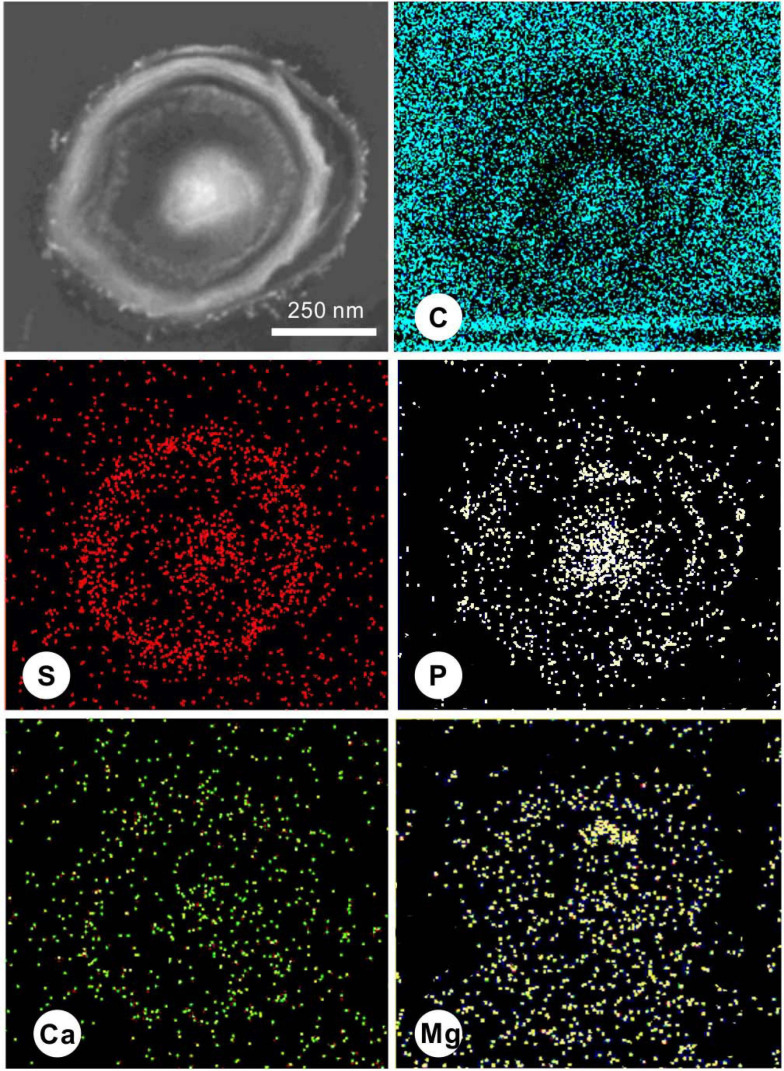
Scanning transmission electron microscopy (STEM) elemental mapping of a 40-nm thin section of a typical *E. ludwigii* SYB1 cell in Nutrient Broth (NB) medium with an Mg/Ca molar ratio of 3 showing the distribution of C, S, P, Ca, and Mg.

During the process of biomineralization, the cells themselves do undergo significant changes. TEM imaging at the same magnifications as ultrathin sections revealed significant differences in the cell surface between those in the seed liquid and those in the experiments. Ultrathin sections of cells from the seed liquid medium (i.e., NB medium without Ca^2+^ and Mg^2+^) were used as the control (Figure 8A). The cells there appeared smooth with no visible coating; the chromatin filaments inside the cell can also be recognized. The multilayered cell-wall structure further proves that they are Gram-negative bacteria. In contrast, most cells in the biomineralization medium (NB medium with Ca^2+^ and Mg^2+^) are encrusted by a large amount of nanoparticles after 2 weeks of incubation (marked by the yellow triangle) (Figures 8B–D). As the medium Mg/Ca molar ratio increases, the number and size of nanoparticles also tend to increase, and a particle with a diameter of about 200 nm appeared on the EPS around the cell from the medium with the highest Mg/Ca ratio (9). The lattice fringes and the fast Fourier transform (FFT) pattern of the largest particle show evidence for the presence of very small but crystalline nanocrystals (Figures 8E,F). The calculated interplanar spacing (2.83 Å) accurately matches with the crystal face (301) of MHC (Figure 8G), indicating the presence of nano-MHC around EPS. EDS analysis also showed the appearance of C, O, and Mg (Si and Cu were coming from the sample holder) (Figure 8H). Hence, it is reasonable to assume that the nuclei of MHC and some other Mg-bearing carbonates were formed on the surface of the EPS. Elemental mapping of a typical thin section further revealed that there was a clear correlation between element distribution and cell structure (Figure 9). Since the ultrathin section is supported by the carbon film, the C element almost fills the entire field of view, but the shape of the cell can still be recognized. The P and S elements are mainly distributed near the cell wall and nucleus area, which is due to the presence of the phospholipid bilayer outside the cell (G− bacteria) and the nucleic acid inside the cell. Metal cations including Ca and Mg elements are significantly enriched on the surface and inside the cells. These results provide a strong evidence for the absorption and nucleation of minerals on the surface of *E. ludwigii* SYB1.

### DFT Adsorption Simulation of Amino Acids on MHC Crystal Faces

**FIGURE 10:**
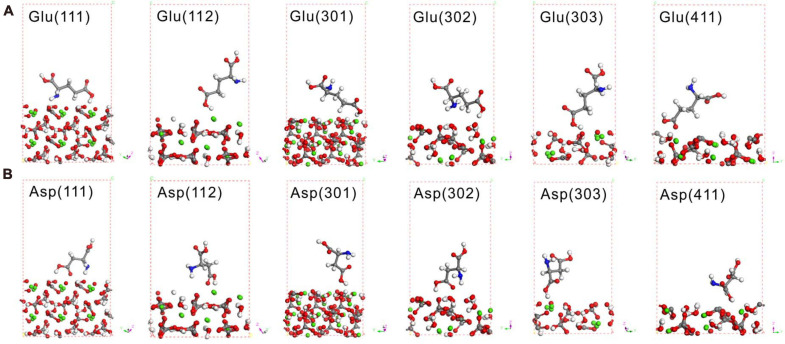
Density functional theory (DFT) simulation of Glu **(A)** and Asp **(B)** on various crystal faces of monohydrocalcite (MHC) after geometry optimization. Red spheres denote O, gray spheres denote C, white denotes H, blue denotes N, and green denotes Ca.

Theoretical calculations adopting DFT can provide reliable interpretations of experimental adsorption data, thus leading to an understanding of the structures and mechanisms of organic molecular adsorption onto mineral surfaces at the atomic scale (Harouaka et al., 2017). The most likely high-symmetry adsorption sites and binding modes were considered and calculated to determine the adsorption of amino acids on the various crystal surfaces of MHC. First, Glu and Asp were taken to calculate their adsorption characteristics on the (222) crystal face of MHC (the preferred orientation face) because they were the two amino acids with the highest content in MHC (Supplementary Figure 6). Before adsorption, Glu was in a prone state on the crystal plane with a contact angle of about 1.5°. After optimizing the structure of the entire adsorption system, it was found that the Glu molecule had been rotated to be about 72°. This shows that the adsorption of the crystal plane shifts the spatial position of amino acids. The calculated adsorption energy of Asp and Glu on the (222) crystal face of MHC was about −0.04811 and −0.0561 eV, respectively. It confirmed the adsorption of amino acids on the (222) crystal face. The spatial geometric position of Glu and Asp on different crystal faces is shown in Figure 10; it could be seen that most of them were at a high contact angle with the crystal face.

**FIGURE 11:**
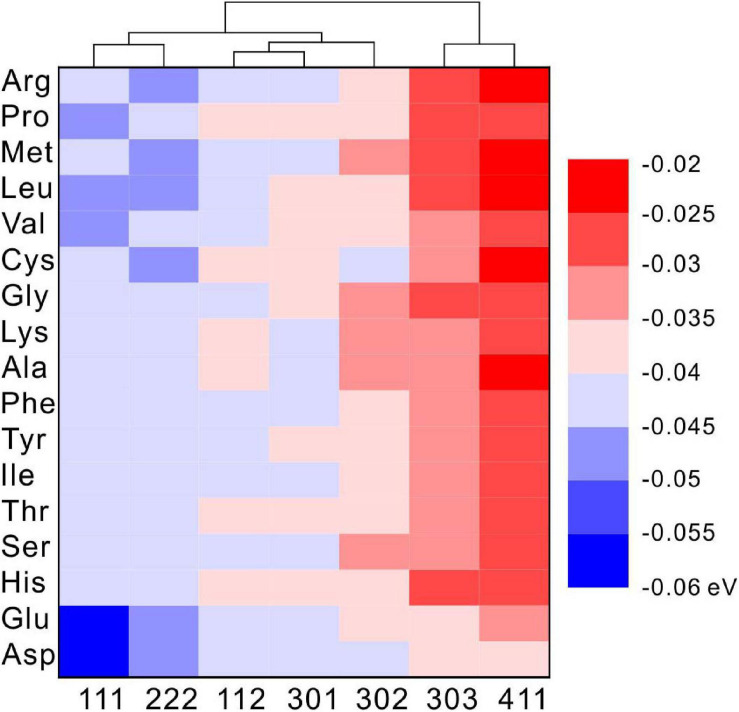
The density functional theory (DFT) calculated adsorption energy of amino acids on different diffraction crystal faces [(111), (112), (301), (302), (303), and (411)] of monohydrocalcite (MHC).

Under selected restrictions, the adsorption energies of 17 kinds of amino acid that appeared in the MHC structure, on the (111), (112), (301), (302), (303), and (411) crystal planes of MHC, are shown in Figure 11. In this configuration, two conclusions can be drawn from the heat map: first, on the same crystal surface, the adsorption energies of Asp and Glu are always the lowest, indicating that they have the strongest adsorption capacity on the surface. Second, the adsorption energy of the same amino acid on different crystal surfaces is also different, suggesting that the different crystal surfaces of MHC themselves have different adsorption capacity.

## Discussion

Given the unique surface texture, elemental composition, and involved organic matters of the minerals observed in the present experiments, it is reasonable to conclude that the facultative anaerobic strain *E. ludwigii* SYB1 strongly affects the formation of carbonate under aerobic culture conditions. This is not unexpected because a variety species of bacteria have been shown to have the ability to induce or control the formation of carbonate minerals, including cyanobacteria, sulfate-reducing bacteria, and halophilic bacteria (Sánchez-Román et al., 2008; Han et al., 2017; Qiu et al., 2017; Zhang et al., 2020). However, the results from the present study point to several new understandings that may shed light on the route and mechanism of bacterially induced carbonate precipitation.

### Alkaline Conditions Induced by SYB1

Among the variable solution conditions, an alkaline state is a prerequisite in the formation process of carbonate minerals in aquatic systems (Lian et al., 2006; Sánchez-Román et al., 2008). It has generally been accepted that microbial degradation of nitrogenous organic compounds (protein, peptide, amino acids, etc.) leads to the alkalization and ammoniation of the medium (Sánchez-Román et al., 2008; Li et al., 2019).

In our present bioreactors, the initial pH conditions of all solutions, including those for the control experiments, are about 7.0. No minerals were formed in the control groups without inoculation of SYB1, definitely indicating that the presence of *E. ludwigii* SYB1 is responsible for the final alkaline condition. The self-mediated pH increase in SYB1 is an interesting phenomenon, since it was found that the measured concentration of ammonia is not enough to achieve the final pH value (about 9.0–9.2) (Figure 2F). The detected increase in pH can be attributed to two reasons. First is the ammoniation of the medium. Ammonia provides part of the alkalinity during hydration (${\text{NH}}_{3}+H_{2}\text{O}\to {\text{NH}}_{4}^{+}+{\text{OH}}^{-}$). Second is the catalysis of CA. The rapid metabolism of bacteria will result in the enrichment of carbon dioxide in the medium, which is detrimental to the formation of minerals. The catalysis of carbonic anhydrase can quickly convert carbon dioxide in the solution, leading to the detectable enhancement of HCO_3_^–^ and CO_3_^2–^ concentrations (Figure 2D). As a result, an alkaline and supersaturated condition can be achieved to permit and maintain the onset of microbial precipitation.

### Coprecipitation of Monohydrocalcite and Dypingite Induced by SYB1

Many studies have confirmed that the formation of MHC requires the presence of magnesium in solution (Mg/Ca ratios ≥ 0.17 < 65) (Rodriguez-Blanco et al., 2014). As for the occurrence state of Mg in chemically synthesized MHC structures, there are at least four different explanations: (1) the spherulitic MHCs were covered by an external layer with a high Mg concentration, which played a protective role for the stability of MHC (Dejehet et al., 1999); (2) the additional Mg in the precipitates was a crystallized discrete phase (maybe nesquehonite with low crystallinity) (Nishiyama et al., 2013); (3) the Mg has infiltrated into the structure of MHC (χ*MgCO*_3_ = 0.26) (Rodriguez-Blanco et al., 2014); and (4) the Mg in MHC is a mixture of amorphous Mg carbonate (AMC) and other Mg-containing phases (Fukushi et al., 2017). In the above chemical synthesis experiments, calcium-free Mg-carbonate minerals are rare. However, in our biotic experiments, a flaky Mg-bearing coating around MHC was observed in a medium with an Mg/Ca ratio of 3, at the same time, a discrete Mg carbonate phase (dypingite) coprecipitated with MHC could also be identified in the medium with higher Mg/Ca ratios. The coating minerals and discrete dypingite are similar in single slice thickness (about 25 nm), morphology, and elemental composition (Figures 4G,H). Based on the results, it is reasonable to hypothesize that the magnesium was released from the MHC crystals and enriched on the surface. Then, they crystallized to single flaky dypingite with low crystallinity, which could detach from the surface of the MHC and continue to grow to form the flower-like dypingite.

The results presented here revealed that the bacterially induced MHC crystallization was different from that of the chemically synthesized MHC. The large number of organic molecules produced by microorganisms may help reduce the kinetic barrier to the precipitation of magnesium ions. The strong interaction between negatively charged organic functional groups and positively charged free metal cations can lead to precipitation (Qiu et al., 2017), and these organic materials could inhibit the transformation from hydrous metastable minerals [CaCO_3_⋅H_2_O, Mg_5_(CO_3_)_4_(OH)_2_⋅5H_2_O] to relatively stable phases (CaCO_3_, MgCO_3_⋅3H_2_O) via a dissolution–recrystallization process. This is confirmed by our FTIR, XPS, and confocal laser scanning microscopy (CLSM) analyses identifying that the organics are indeed occluded in the structure of the biominerals. Therefore, the coprecipitation of MHC and dypingite demonstrates that various organic components cannot only induce the formation of carbonate minerals via the chelation process but also stabilize the hydrous metastable mineral phase.

### Adsorption of Amino Acids on MHC Crystal Planes

In the EPS of SYB1, 17 kinds of amino acids were identified, which were similar to previous studies (Qiu et al., 2017; Zhuang et al., 2018; Zhao et al., 2019). However, the content of various types of amino acids in MHC and EPS is quite different. In the EPS, the molar percentage of Glu, Gly, and Asp are significantly higher than other types of amino acids. In contrast, the percentage of Glu and Asp in MHC is significantly higher than other types of amino acid. This indicates that Glu and Asp can preferentially enter the structure of MHC. Glu and Asp are acidic amino acids containing two carboxyl groups. One of the carboxyl groups remains free after dehydration and condensation and can preferentially bind to free metal cations (Qiu et al., 2017), thereby they could preferentially be incorporated with minerals. However, there is also another possibility that the high concentrations of Glu and Asp in the EPS permit them to enter the relatively open MHC crystals through free diffusion instead of adsorption. If so, the proportion of Glu and Asp in MHC will not exceed their proportion in the EPS. However, they are obviously more concentrated than those in the EPS, with a significant difference (*p* < 0.5) (Figure 7). At the same time, the content of Gly is significantly lower than that in the EPS, suggesting that there is competitive adsorption between different amino acids. Moreover, DFT simulated calculations have further confirmed that, compared to other types of amino acids, Glu and Asp always have the lowest adsorption energy on seven diffracted crystal planes of MHC, indicating that they have the stronger binding ability with MHC. Therefore, in our case, it is confirmed that Glu and Asp were preferentially incorporated into the MHC structure through the adsorption process.

On the other hand, the cluster analysis of each crystal plane showed that various amino acids always have lower adsorption energies on the (111) and (222) crystal planes. The simulation results indicated that various amino acids tend to be adsorbed on these two crystal planes. Studies have shown that the adsorption of organic components on the crystal surface of minerals can reduce the normal growth rate of the crystal surface, thereby changing the final morphology of the crystal (Cody and Cody, 1991; DeOliveira and Laursen, 1997; Wang Z. et al., 2015; Harouaka et al., 2016). According to the Bravais rule, the crystal planes with fast growth rate tend to disappear, while the crystal planes with slow growth rate tend to remain on the crystal. Therefore, in this study, the growth inhibition of (111) and (222) crystal planes by amino acids slowed down their growth rate and made them more likely to appear on the outside of the crystal. Since the (111) crystal plane was originally the crystal plane with the strongest intensity (PDF card #29-0306 in Jade 6.0), the preferred orientation was not shown on the XRD data. Our results further showed that amino acids cannot only promote nucleation by negatively adsorbing metal cations, but they can also be mixed into the MHC crystal structure and so affect the growth and structure of the crystal.

### Proposed Model of Biomineralization Induced by *E. ludwigii* SYB1

To sum up the experimental observations, we propose the following model for *E. ludwigii* SYB1-induced carbonate biomineralization. In the initial stage of bacterial culture, the pH of the medium is about 7.0, and carbonate minerals cannot be precipitated under this condition. With the rapid growth of SYB1, ammoniation [Equations (1,2)] and the catalysis of carbonic anhydrase [Equations (3,4)] cause the solution to transform from unsaturated to supersaturated relative to carbonate minerals. The transformation process of CO_2_ and –NH_2_ functional groups in amino acids can be represented as follows:

       
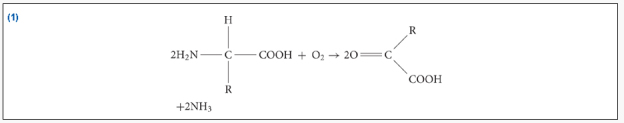


(2) $${\text{NH}}_{3}+H_{2}\text{O}\to {\text{NH}}_{4}^{+}+{\mathrm{OH}}^{-}$$

(3) $${\text{CO}}_{2}+H_{2}\text{O}↔{\text{HCO}}_{3}^{-}+H^{+}$$

(4) $${\text{HCO}}_{3}^{-}+{\mathrm{OH}}^{-}↔{\text{CO}}_{3}^{2-}+H_{2}\text{O}$$

In the process, the organic functional groups such as carboxylic acid in the extracellular polymers of the bacteria deprotonate and interact strongly with the free metal cations, and the mineral saturation near the bacteria is further increased. Our EDS results showed that a large amount of calcium and magnesium ions are enriched on the surface of the bacteria. Since the bacteria have been thoroughly cleaned three times by a phosphate buffer solution before the preparation of the ultrathin slices, it indicates that there is a strong chelation between the EPS and metal ions. Then, the nucleation and growth of minerals have started. A series of studies have shown that carbonate minerals are formed by the gradual aging and crystallization of amorphous precursors under the action of a large number of microorganisms, such as the process of the *Lysinibacillus* sp. GW-2 strain inducing vaterite (Lv et al., 2017). In our study, the selected area electron diffraction showed that the irregular particles with lattice fringes on the surface of the EPS were enveloped by amorphous substances, which implicates a process of gradual precipitation of crystal nuclei from the amorphous structure. Once the crystal nucleus is formed, the growth of the crystal will accelerate. The amino acids present in the EPS will be adsorbed on each crystal face and enter the inside of the mineral, thereby leading to crystal plane dislocation and preferred orientation based on these crystal nuclei templates (Qiao et al., 2008; Olson et al., 2013).

It should be noted that, when facultative anaerobes are in an anaerobic environment, their mineralization process may be different. This will be explored in-depth in follow-up research and compared with the results presented here.

### Implications for the Formation of Natural Minerals

The coprecipitation of MHC and DYP in the present biomineralization experiments is significant because similar assemblages of mineral also occur in modern fresh or saline lake environments and in young sediments (Last, 1992; Goto et al., 2003; Hayakawa et al., 2003; Solotchina et al., 2009; Last et al., 2010; Li et al., 2012). However, the presence of MHC and/or DYP in the sedimentary record is rare, whereas abundant anhydrous Ca–Mg carbonates are present in those same ancient rocks. MHC and DYP are metastable carbonate phases; they can be altered, dissolved, and/or transformed to the more stable carbonate phases including calcite, Mg-calcite, and magnesite. Thus, it is reasonable to hypothesize that there are some carbonate deposits found in the geological record that originally formed via a bacterially induced metastable intermediate.

The present results indicate a relationship between facultative anaerobic bacterial activity and precipitation of MHC and DYP. These carbonates could be mediated by facultative anaerobic bacteria in a wide range of environments with similar Mg/Ca ratios as studied in this research. The enriched organic materials, including amino acids, in these carbonate minerals can be regarded as one of the biosignatures when they appeared simultaneously with the microbially influenced sedimentary structures and the morphological biosignatures (Cady et al., 2003; Summons et al., 2011).

## Conclusion

This study shows that facultative anaerobic *E. ludwigii* SYB1 bacteria cultured in the aerobic medium can promote the alkalization and saturation of the solution through ammonization and the CA activity. Calcium and magnesium were concentrated around the cell by the adsorption of the negatively charged organics onto the EPS; minerals then nucleate and grow from amorphous precursors on the surface of the bacteria and the EPS. In the growth of minerals, it was found that among the 17 kinds of amino acids, Glu and Asp can preferentially enter the structure of MHC. Various types of amino acids tend to be adsorbed on specific diffracted crystal planes, which may cause the development of complex crystal morphologies and preferred orientations. The present results indicate that MHC and DYP in the natural environment may be related to the activity of facultative anaerobic bacteria, and amino acids in these minerals can serve as one of the biosignatures to recognize their bacterial origin.

## Data Availability Statement

The datasets generated for this study can be found in online repositories. The names of the repository/repositories and accession number(s) can be found below: https://www.ncbi.nlm.nih.gov/, MW266032.1.

## Author Contributions

YZ, HY, and HZ did the isolation and culture of SYB1. ZH and HY organized the laboratory experiments. YZ carried out the biomineralization experiments. XG and RM carried out the XRD analysis. NG and DO carried out the FTIR and SEM analysis. MT helped to draft the manuscript. All authors read and approved the final manuscript.

## Conflict of Interest

The authors declare that the research was conducted in the absence of any commercial or financial relationships that could be construed as a potential conflict of interest.

## Publisher’s Note

All claims expressed in this article are solely those of the authors and do not necessarily represent those of their affiliated organizations, or those of the publisher, the editors and the reviewers. Any product that may be evaluated in this article, or claim that may be made by its manufacturer, is not guaranteed or endorsed by the publisher.
