# Supplementary material for: Selective Adsorption of Amino Acids in Crystals of Monohydrocalcite Induced by the Facultative Anaerobic Enterobacter ludwigii SYB1
Source: Front Microbiol. 2021 Jul 29;12:696557. doi: 10.3389/fmicb.2021.696557 (PMC8358455; doi:10.3389/fmicb.2021.696557)
Supplement: Supplementary file 1 [file Data_Sheet_1.docx]

Supplementary Material

**Supplementary Table 1** The physiological comparison of SYB1 and typical *Enterobacter ludwigii*

| Experimental item | SYB1 | Standard Enterobacter ludwigii |
| --- | --- | --- |
| V-P | + | + |
| Hydrogen sulfide | + | + |
| Inclined plane | - | - |
| Three usgar iron Bottom | + | + |
| Hydrogen sulfide | - | - |
| Gas production | + | + |
| Starch hydrolysis | - | - |
| Methyl red | - | - |
| Citric acid salt | + | + |
| Cellulose | - | - |
| Indole | + | + |
| Motility | + | + |
| Lipase | - | - |
| Ammonia production | + | + |
| Catalase  Gram staining | +  - | +  - |


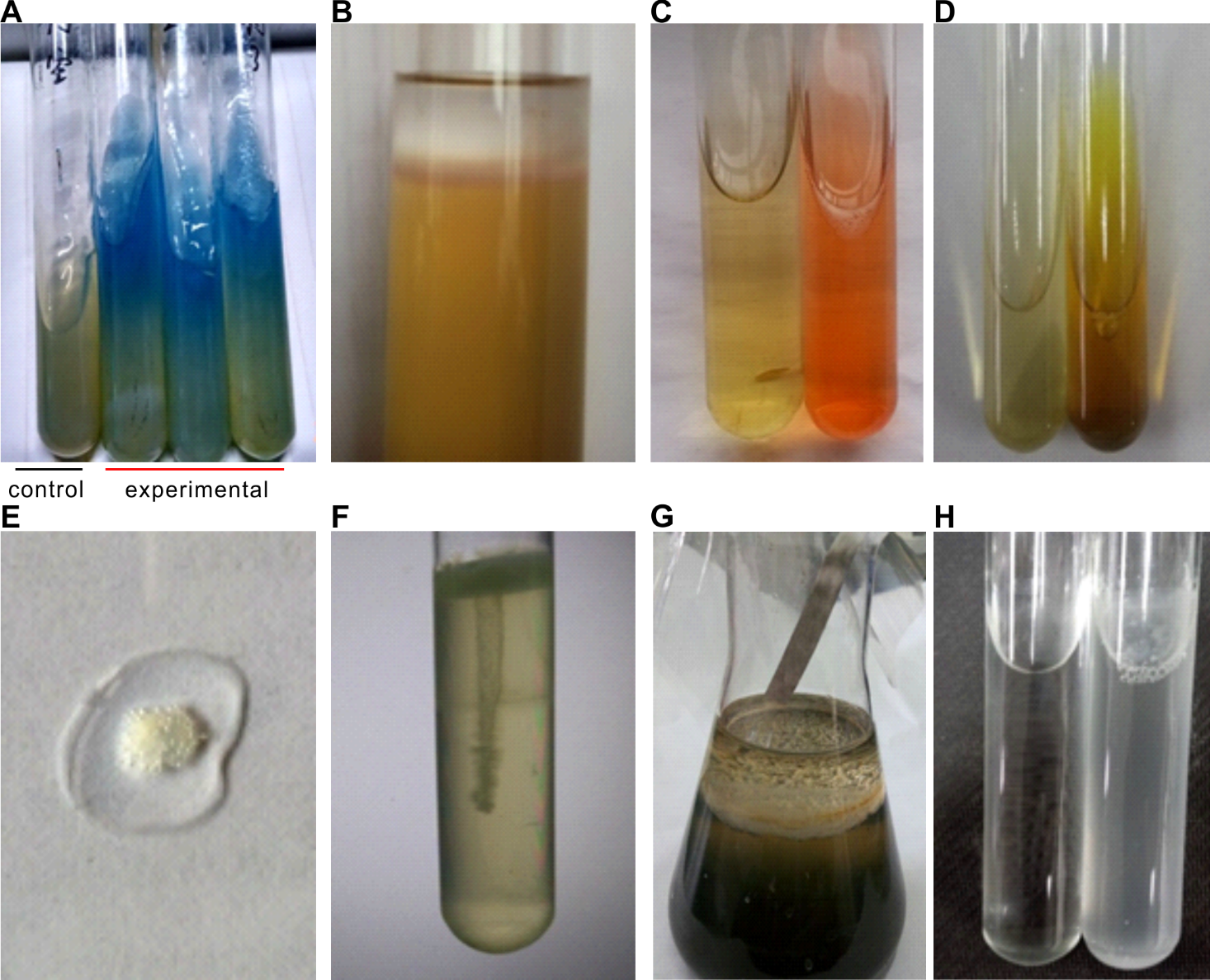


**Supplementary Figure 1.** Some positive physiological reactions of SYB1. (**A**) Citric acid salt, (**B**) Indole, (**C**) V-P, (**D**) Ammonia production, (**E**) Catalase, (**F**) Motility, (**G**) Hydrogen sulfide production, (**H**) Sole ammonium nitrogen source.


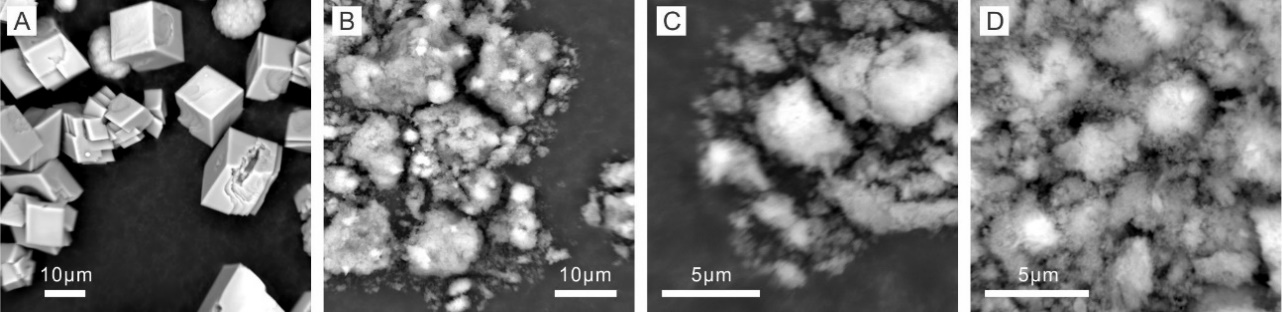


**Supplementary Figure 2.** Precipitates in biomimetic groups with the various Mg/Ca molar ratio. **(A)** Mg/Ca=0, **(B)** Mg/Ca=3, **(C)** Mg/Ca=6, **(D)** Mg/Ca=9.


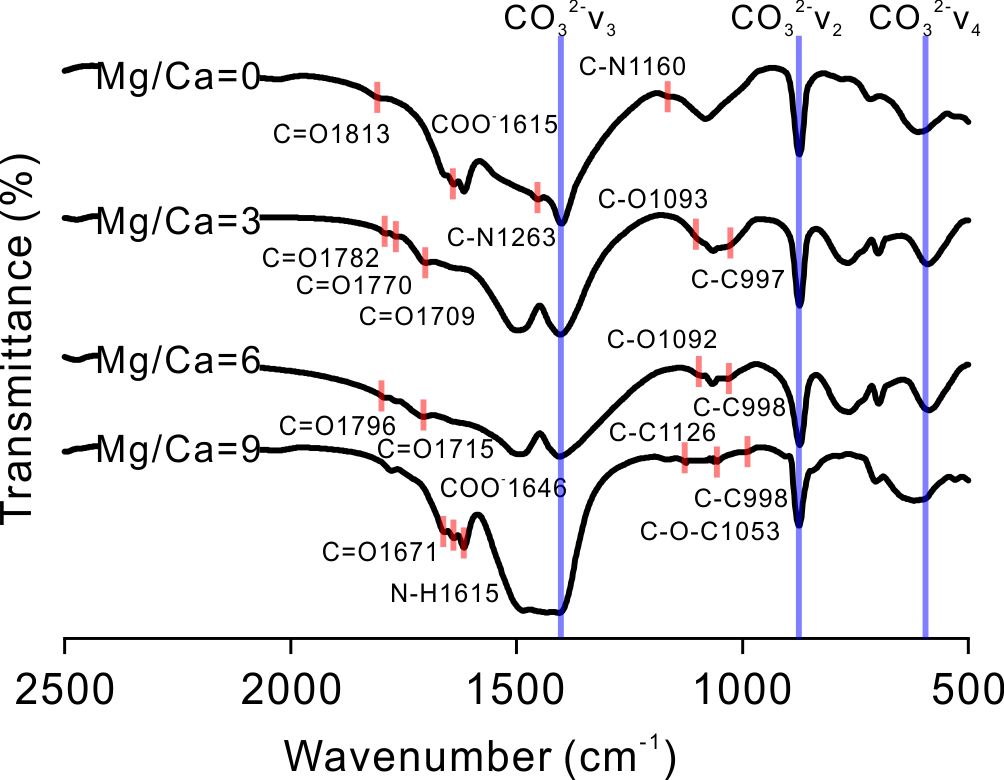


**Supplementary Figure 3.** FT-IR spectrums of the biominerals induced by *E. ludwigii* SYB1 in NB media with different Mg/Ca molar ratios. For inorganic functional groups, only ν_2_, ν_3_ and ν_4_ bands of CO_3_^2-^ were labeled.


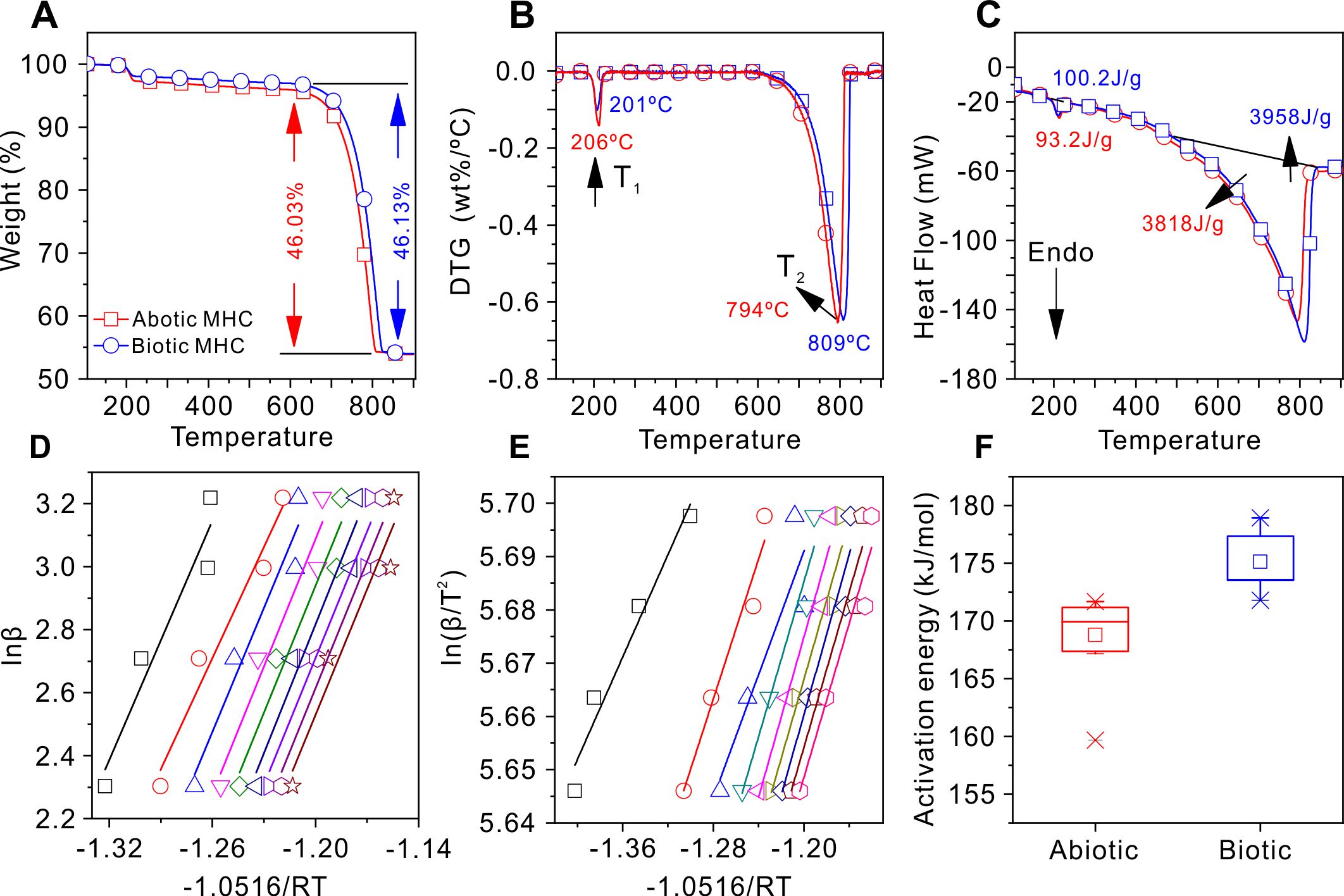


**Supplementary Figure 4.** The comparation of TG-DSC analysis showing higher thermal stability of bio-induced MHC compared to that of chemical synthesized MHC. (A-C) TG, DTG and DSC curves of biotic and abiotic MHC; (D) activation energy of biotic MHC based on FWO method; (E) activation energy of abiotic MHC based on KAS method; (F) comparation of activation energy of biotic and abiotic MHC.


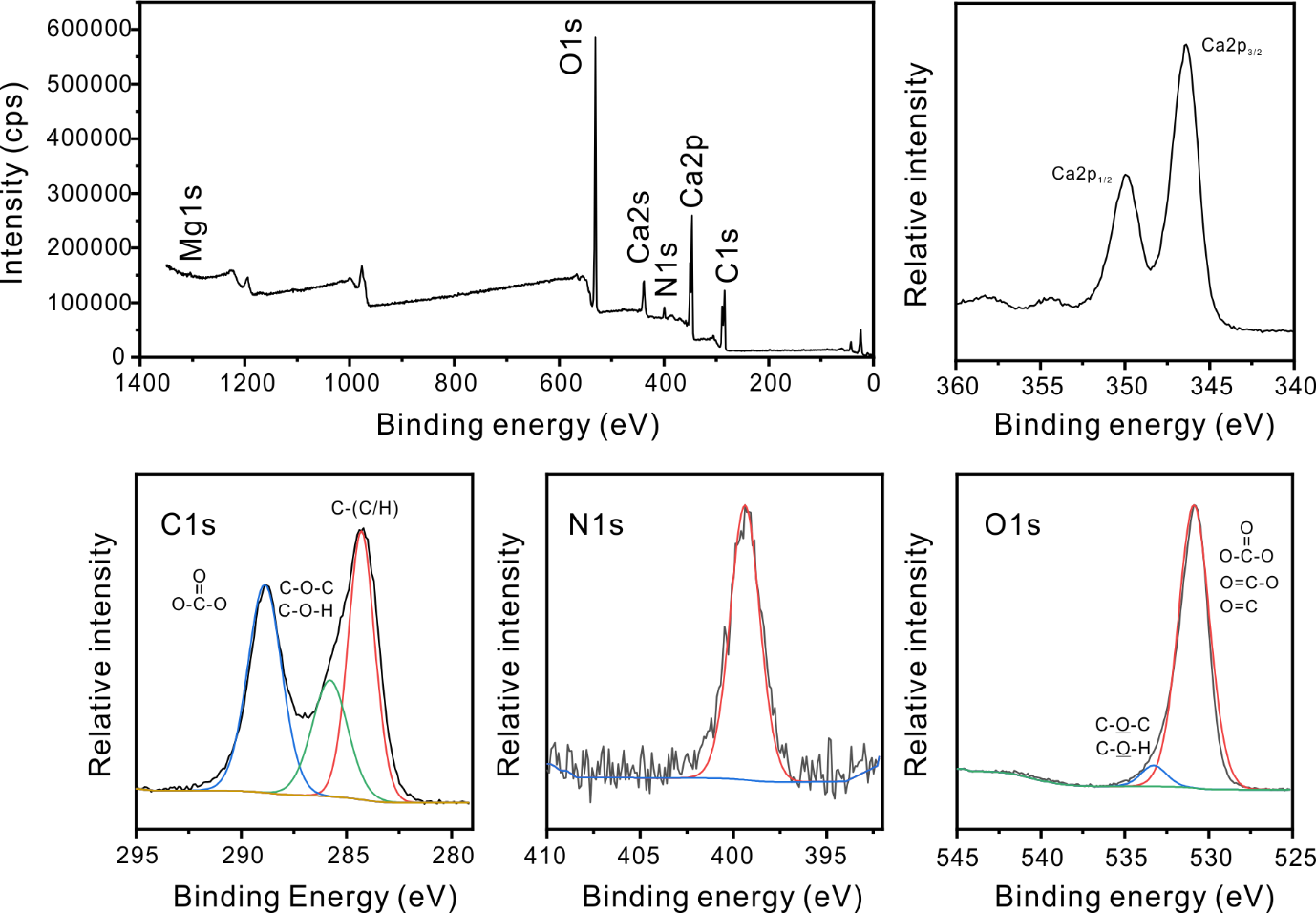


**Supplementary Figure 5.** XPS analysis of MHC crystals induced by E. ludwigii SYB1 in medium with Mg/Ca ratio of 3.


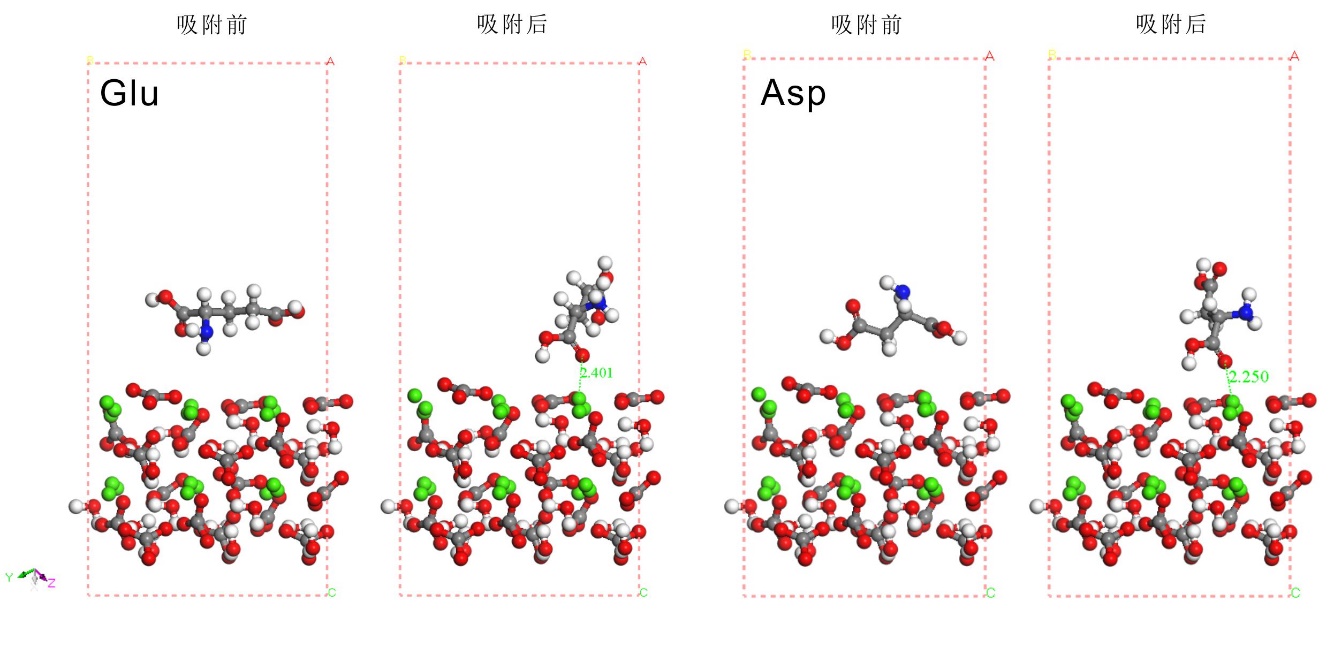


**Supplementary Figure 6.** Spatial position of Glu and Asp on (222) crystal faces before and after geometry optimization.
